# Statistical inference for a quasi birth–death model of RNA transcription

**DOI:** 10.1186/s12859-022-04638-6

**Published:** 2022-03-26

**Authors:** Mathisca de Gunst, Michel Mandjes, Birgit Sollie

**Affiliations:** 1 Department of Mathematics, Vrije Universiteit Amsterdam, de Boelelaan 1111, 1081 HV Amsterdam, The Netherlands; 2 Korteweg-de Vries Institute for Mathematics, University of Amsterdam, Science Park 904, 1098 XH Amsterdam, The Netherlands; 3 Eurandom, Eindhoven University of Technology, Eindhoven, The Netherlands; 4 Amsterdam Business School, Faculty of Economics and Business, University of Amsterdam, Amsterdam, The Netherlands; 5 Department of Epidemiology and Data Science, Amsterdam UMC, Vrije Universiteit Amsterdam, de Boelelaan 1117, 1081 HV Amsterdam, The Netherlands

**Keywords:** Quasi birth–death process, Maximum likelihood estimation, Erlangization technique, RNA transcription

## Abstract

**Background:**

A birth–death process of which the births follow a hypoexponential distribution with *L* phases and are controlled by an on/off mechanism, is a population process which we call the on/off-seq-*L* process. It is a suitable model for the dynamics of a population of RNA molecules in a single living cell. Motivated by this biological application, our aim is to develop a statistical method to estimate the model parameters of the on/off-seq-*L* process, based on observations of the population size at discrete time points, and to apply this method to real RNA data.

**Methods:**

It is shown that the on/off-seq-*L* process can be seen as a quasi birth–death process, and an Erlangization technique can be used to approximate the corresponding likelihood function. An extensive simulation-based numerical study is carried out to investigate the performance of the resulting estimation method.

**Results and conclusion:**

A statistical method is presented to find maximum likelihood estimates of the model parameters for the on/off-seq-*L* process. Numerical complications related to the likelihood maximization are identified and analyzed, and solutions are presented. The proposed estimation method is a highly accurate method to find the parameter estimates. Based on real RNA data, the on/off-seq-3 process emerges as the best model to describe RNA transcription.

## Background

Birth–death (bd) processes are continuous-time Markov processes with two types of transitions; *births* which increase the state by one, and *deaths* which decrease the state by one. bd processes are suitable for modelling the dynamics of the number of individuals in a population, and are widely used in a broad range of areas such as biology, ecology and operations research. The research in this paper is motivated by a specific biological application: the number of RNA molecules in a single living cell. The evolution of a population of RNA molecules can be modelled by a bd process, since the population can increase (production) or decrease (degradation) by one molecule at a time. A complication, however, is that it is known that the production of RNA molecules is a sequential process consisting of multiple phases [8, 13], and that the production is regulated by an on/off mechanism [10], which we will refer to as the on/off switch. To model the population of RNA molecules in a realistic way, we therefore extend the basic bd process by including these two features to the model. This results in what we call the on/off-seq-*L* process, which is also considered in [3]. The on/off switch in the on/off-seq-*L* process is a mechanism that decides if the next birth of an individual can be set in motion or not. Births can be initiated only while the switch is turned on. If the switch turns off, it needs to be switched back on before a birth can be initiated. Once a birth has been initiated, it takes *L* sequential independent exponentially distributed phases before a new individual is born and the population increases by one.

Our objective is to develop a statistical inference method for the on/off-seq-*L* process, which we wish to apply to a real data set of RNA counts in cells. In line with the structure of our real data set, we focus on a setting in which we have access to longitudinal measurements on the number of RNA molecules in a large number of independent cells. The concrete goal is to estimate the model parameters based on observations of the population size at discrete time points, and to perform model selection on the on/off switch and on the number of phases *L* in the birth process. This kind of inference problem has been studied before in the context of RNA transcription. We mention [5], where maximum likelihood estimates are computed and a model selection procedure is performed for a stochastic model with a sequential birth process. However, in contrast to the on/off-seq-*L* process, an on/off mechanism is not included in that model. In [3, 9], maximum likelihood estimation and a model selection procedure are performed for the on/off-seq-*L* process. However, in these studies the likelihood function is computed from observations of the transcription intervals, that is, the time between two consecutive RNA births. These intervals are not known exactly, since the data is interval censored. In the present paper, we use a method to evaluate the likelihood function from observations of the population size, instead of the transcription intervals. To this end we make use of the fact that the on/off-seq-*L* process can be seen as a quasi birth–death (qbd) process.

A quasi birth–death (qbd) process is a bd process of which the transition rates are affected by an underlying continuous-time Markov chain, often referred to as the phase process. Together, the population process and the phase process form a bivariate Markov process. The class of qbd processes owes its popularity to the fact that it is comprehensive (in that it is capable of accurately approximating rather general population processes), while at the same time it allows for explicit calculations. Various properties of qbd processes have been studied over the years: we refer to [2] for calculations of the equilibrium distribution, to [11] for properties of specific relevant rate matrices, and to [7] for a study on the distribution of the running maximum of the process.

To perform statistical inference, we need sound methodology to compute the likelihood function from observations of the population process. This, in turn, requires techniques for the evaluation of the time-dependent probabilities corresponding to qbd processes, which is a challenging task due to the hidden, unobserved elements of the model. These challenges are discussed in detail in [6], where a method is presented to numerically approximate the time-dependent distribution of the bivariate Markov process of a qbd process. More specifically, [6] proposes, and formally justifies, an approach based on the so-called Erlangization technique. This technique, which has been studied in other contexts as well [1, 7, 12], exploits the fact that, although it may be computationally hard to evaluate the distribution of the state of the bivariate Markov process at a deterministic time, it *can* be computed at exponentially distributed epochs relatively easily. Using the fact that one can approximate a deterministic number arbitrarily closely by the sum of exponentially distributed numbers, one can thus obtain accurate approximations of the distribution of the qbd at deterministic epochs. In this paper we rely on the Erlangization technique as developed in [6] to evaluate the likelihood function from observations of the population size.

The remainder of this paper is organized as follows. In “Mathematical model and estimation problem” section, we mathematically define the on/off-seq-*L* process and introduce the corresponding likelihood function and estimation problem. “Quasi birth–death framework” section shows that the on/off-seq-*L* process belongs to the class of qbd processes, and therefore the Erlangization method as introduced in [6] can be used to approximate the likelihood. By an extensive numerical study in “Numerical study” section, we investigate the accuracy of the resulting estimation method for the on/off-seq-*L* process. In addition, we explore numerical complications related to the likelihood maximization. “RNA transcription” section describes in detail the biological process of RNA transcription, which is the motivating application of this paper. A model selection procedure is performed for different on/off-seq-*L* processes, based on data of RNA counts in single cells. The paper is concluded by a discussion in “Discussion” section.

## Methods

### Mathematical model and estimation problem

In this section we formally introduce the class of on/off-seq-*L* processes together with the necessary notation. We then define the estimation problem and the corresponding likelihood function.

#### The on/off-seq-*L* process

The on/off-seq-*L* process can be viewed as a bd process with two specific features in the birth process. First, the births follow a hypoexponential distribution—that is a sum of exponentially distributed phases—instead of the often used exponential distribution. Second, the births are controlled by a so-called on/off switch, which means that births can be initiated only while the switch is turned on. Because of this specific structure, the on/off-seq-*L* process is modelled as a two-dimensional Markov process, consisting of the population process together with an underlying background process. We start with the mathematical definition of this background process, which can be viewed as a process that keeps track of the status of the birth process. We then define the population process and complete the definition with the two-dimensional Markov process and its transition rates.

**Fig. 1:**
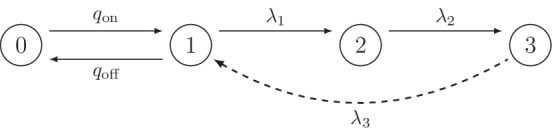
Schematic representation of the $\{X_t\}$ process in the on/off-seq-3 model. The dotted line indicates the transition that results in a birth of a new individual. Parameters $q_{{{\mathrm{off}}}}, q_{{{\mathrm{on}}}}, \uplambda _1, \uplambda _2$ and $\uplambda _3$ denote the transition rates

Let {*X*_*t*_}_*t* ≥ 0_ be a continuous-time Markov chain modeling both the on-off switch of the process and the exponential phases of the birth process. Its state space is given by $E = \{0,1,\ldots ,L\}$. We assume that the distribution of $X_0$, the initial state distribution, is equal to the (unique) stationary distribution of $\{X_t\}$. The state $X_t = 0$ corresponds to the state where the on/off switch is turned off, and will be referred to as the off-state. Importantly, births cannot be initiated in this state. The switch needs to switch back on first, leading to the state $X_t = 1$, which we refer to as the on-state. Births can only be initiated from this state. Once a birth is initiated, the process runs through states $1,\ldots ,L$ and back to state 1, corresponding to the sequential, exponential phases of the birth process. A schematic representation is given in Fig. 1 for the model with $L = 3$. When the *L* exponential phases are completed, a new individual is born and the population increases by one. During this birth process, the switch remains on.

Let $\{M_t\}_{t\ge 0}$ be the population process, with $M_t$ equal to the total number of individuals in the system at time *t*. The birth process that increases the population size is described above. The population size decreases according to a general death process, where the lifetimes of the individuals are assumed to follow an exponential distribution, independently of each other, and independently of $\{X_t\}$. The entire model is described by the two-dimensional, time-homogeneous Markov process $\{X_t, M_t\}_{t\ge 0}$. Combining the definitions of $\{X_t\}$ and $\{M_t\}$, we can define the transition rates of this joint process.

First, we have the two rates associated with the on–off mechanism. These rates correspond to jumps of $\{X_t\}$ between states 0 and 1 while the state of $\{M_t\}$ remains unchanged. When $M_t = m$, we have, for all $m\ge 0$, the transition rate $q_{{{\mathrm{on}}}}$ for the transition from (0, *m*) to (1, *m*) and the rate $q_{{{\mathrm{off}}}}$ for the transition from (1, *m*) to (0, *m*). Note that $q_{{{\mathrm{on}}}}$ and $q_{{{\mathrm{off}}}}$ do not depend on *m*. Secondly, we have the rates associated with the sequential birth phases, where the state of $\{M_t\}$ remains unchanged until the completion of the final phase. For all $L \ge 2$ we have rates $\uplambda _i$ for the transitions from (*i*, *m*) to $(i+1,m)$, $i \in {1,\ldots ,L -1}$, and for all $L \ge 1$ we have rate $\uplambda _L$ for the transition (*L*, *m*) to $(1,m+1)$. Note that after completion of the final phase, the process $\{X_t\}$ returns to state 1 from which the system can either be turned off, or a new birth can be initiated. Last, we have the rates associated with the deaths. The lifetimes of the individuals follow an exponential distribution with parameter $\mu$, independently of each other. This means that the total death rate is proportional to the total number of individuals in the population. Furthermore, the lifetimes are not affected by the state of $\{X_t\}$. Hence for all $i \in {1,\ldots ,L}$ and $m > 0$, we have rate $m\mu$ for the transition (*i*, *m*) to $(i,m-1)$.

#### Likelihood evaluation

We combine all model parameters of the on/off-seq-*L* process in the parameter vector $\theta = (q_{\text {on}}, q_{\text {off}}, \uplambda _1, \ldots , \uplambda _L, \mu )^\top$. As mentioned above, the goal is to estimate $\theta$ based on observations of the population size at discrete time points, and to perform model selection on the on/off switch and on the number of phases *L* in the birth process. To find maximum likelihood estimates, we need a reliable method to evaluate the likelihood function of the data with respect to $\theta$.

The available data set consists of multiple times series corresponding to *N* independent experiments. Let $\Delta >0$ be the time between two consecutive observations, and let $n+1$ be the number of observations in a single experiment corresponding to observation times $0,\Delta ,2\Delta ,\ldots ,n\Delta$. We assume that in each experiment the process $\{M_t\}$ is observed at these observation times, resulting in observations $m^{(k)}_0,\ldots ,m^{(k)}_n$ for experiments $k = 1,\ldots N$. We introduce the corresponding data vectors $m_{0,n}^{k} = (m_0^{(k)},\ldots , m_n^{(k)})^\top$, $k = 1,\ldots N$. The loglikelihood function based on the *N* independent experiments is then equal to

1 $$\begin{aligned} \log {\mathcal {L}}(\theta \,|\, m^{(1)}_{0,n},\ldots ,m^{(N)}_{0,n} ) = \sum _{k=1}^N \log {\mathcal {L}}(\theta \,|\, m^{(k)}_{0,n}). \end{aligned}$$

We can rewrite the likelihood function, ${\mathcal {L}}(\theta \,|\, m^{(k)}_{0,n})$, for a single data vector $m_{0,n}^{(k)}$, by conditioning on the states of the background process $\{X_t\}$ at the observation times. To this end, we define the transition probabilities

$$\begin{aligned} p_{xx'}(m,m';t) = {{{{\mathbb{P}}}}}(M_t = m', X_t = x' \,|\, M_0 = m, X_0 = x). \end{aligned}$$

Then

2 $$\begin{aligned} {\mathcal {L}}(\theta \,|\, m^{(k)}_{0,n}) = \sum _{x_0,\ldots ,x_n \in E} {{{{{\mathbb {P}}}}}(M_0 = m_0^{(k)}, X_0 = x_0)} \prod _{i=1}^n p_{x_{i-1}x_i}(m_{i-1}^{(k)},m_i^{(k)};\Delta ). \end{aligned}$$

##### *Remark 1*

Expressions (1) and (2) can easily be generalized in case the number of observations *n* is not equal among all experiments. In that case, define the sequence $n_1,\ldots ,n_N$, and replace *n* by $n_k$.

In the next section we show that the on/off-seq-*L* process can be seen as a qbd process. This means that the Erlangization technique as introduced in [6] can be applied to approximate the transition probabilities in (2), and hence the likelihood function (1).

We proceed by sketching the main ideas behind the Erlangization technique; for details we refer to [6]. We first note that for an exponentially distributed time with mean $\eta ^{-1}$, denoted by $T_\eta$, the transition probabilities

$$\begin{aligned} p_{xx'}(m,m';T_\eta ) = {{{{\mathbb{P}}}}}(M_{T_\eta } = m', X_{T_\eta } = x' \,|\, M_0 = m, X_0 = x) \end{aligned}$$

can be computed relatively easily, namely by solving a system of linear equations (which can be done using standard numerical software). We then define the matrix $\Pi _\eta$ as the transition probability matrix whose entries are the $p_{xx'}(m,m';T_\eta )$, for $x,x'\in E$ and $m,m'\ge 0$. A next observation is that for $T_{\eta ,i}$, $i=1,2, \ldots$, denoting a sequence of independent exponentially distributed random variables with mean $\eta ^{-1}$, it holds that

$$\begin{aligned} \sum _{i=1}^{\ell } T_{{\ell }/\Delta ,i}\rightarrow \Delta ,\quad \hbox {almost surely, as}\,\ell \rightarrow \infty . \end{aligned}$$

In words this means that the sum of appropriately scaled exponential random variables, which has an Erlang distribution, converges to a constant. As a consequence, the entries of the $\ell$-step transition probability matrix $(\Pi _{{\ell }/\Delta })^{\ell }$ converge, as ${\ell }\rightarrow \infty$, to the probabilities $p_{xx'}(m,m';\Delta )$ that we are interested in. The idea of Erlangization is to approximate the $p_{xx'}(m,m';\Delta )$ by the entries of $(\Pi _{{\ell }/\Delta })^{\ell }$ for a sufficiently large value of ${\ell }$. In [6] the accuracy of this technique is assessed in detail, and in particular it is pointed out how an appropriate value of ${\ell }$ can be selected.

A technical requirement for application of the Erlangization technique is that the population size $M_t$ is bounded from above by a constant $C\in {{\mathbb {N}}}$. By the nature of the bd process, the state of $M_t$ can only increase by one at a time. This means that for any small constant $\varepsilon >0$, we can choose a constant *C* large enough to ensure that for all $x, x' \in E$ and $m_i^{(k)} < m'$, $k=1,\ldots , N$, $i=1,\ldots ,n$, the transition probability $p_{x x'}(m_i^{(k)},m';\Delta )$ is negligible for $m'>C$, in the sense that

3 $$\begin{aligned} \max _{m'>C} p_{x x'}(m_i^{(k)},m';\Delta ) <\varepsilon . \end{aligned}$$

Hence, we can indeed bound the population size by this constant *C*. How to choose *C* depends on the application at hand. Evidently, the smaller the desired $\varepsilon$, the larger the value of *C* that is needed.

### Quasi birth–death framework

In this section we show that the on/off-seq-*L* process belongs to the class of qbd processes, using the framework as described in [6]. As argued in the previous section, we can assume that the population process $\{M_t\}$ attains values in $\{0,1,\ldots ,C\}$ for some $C>0$.

Let, as in [6], $Q^{(m)}$, $m=0,\ldots ,C$, be the transition rate matrix on state space $E = \{0,1,\ldots ,L\}$, of which the elements correspond to the jumps from $X_t = i$ to $X_t = j$ while the state $M_t = m$ remains unchanged. The diagonal elements of $Q^{(m)}$ are such that the row sums are zero. Note that, in the setting of this paper, $Q^{(m)}$ is actually independent of *m*. For example, for $L = 3$ and all $m\in \{0,1,\ldots ,C\}$, we have

$$\begin{aligned} Q^{(m)} = \begin{pmatrix} -q_{\text {on}} &{} q_{\text {on}} &{} 0 &{} 0 \\ q_{\text {off}} &{} -q_{\text {off}} - \uplambda _1 &{} \uplambda _1 &{} 0 \\ 0 &{} 0 &{} -\uplambda _2 &{} \uplambda _2 \\ 0 &{} 0 &{} 0 &{} 0 \end{pmatrix}. \end{aligned}$$

Next, we introduce the matrix $\Lambda ^{(m)}$ on *E*, of which the elements correspond to the jumps that increase $M_t$ by one, while $X_t$ jumps from state *i* to *j*. Note that for the on/off-seq-*L* process, all $\uplambda _{ij}^{(m)}$ are zero except for the one corresponding to the completion of the final phase of the birth process (if $m \le C-1$). Hence for $L = 3$, and $m \le C-1$, we have

$$\begin{aligned} \Lambda ^{(m)} = \begin{pmatrix} 0 &{} 0 &{} 0 &{} 0 \\ 0 &{} 0 &{} 0 &{} 0 \\ 0 &{} 0 &{} 0 &{} 0 \\ 0 &{} \uplambda _3&{} 0 &{} 0 \end{pmatrix}. \end{aligned}$$

At last, we introduce the matrix ${\mathcal {M}}^{(m)}$ on *E*, of which the elements correspond to the jumps that decrease $M_t$ by one, while $X_t$ jumps from state *i* to *j*. Deaths leave the state of the background process unchanged, hence all $\mu _{ij}^{(m)}$ are zero for $i \ne j$. We have

$$\begin{aligned} {\mathcal {M}}^{(m)} = \begin{pmatrix} m \mu &{} 0 &{} 0 &{} 0 \\ 0 &{} m \mu &{} 0 &{} 0 \\ 0 &{} 0 &{} m \mu &{} 0 \\ 0 &{} 0 &{} 0 &{} m \mu \end{pmatrix}. \end{aligned}$$

We observe that we can write down the transition rate matrix of the joint process $\{X_t, M_t\}$ in terms of the matrices $Q^{(m)}$, $\Lambda ^{(m)}$ and ${\mathcal {M}}^{(m)}$ in the same way as in [6]. The total number of states of $\{X_t, M_t\}$ is $D = (L+1)(C+1)$, and the $D \times D$ transition matrix is equal to

$$\begin{aligned} Q =\left( \begin{array}{cccccc}{\bar{Q}}^{(0)}&{}\Lambda ^{(0)}&{}0&{}\cdots &{}0&{}0\\ {\mathcal {M}}^{(1)} &{}{\bar{Q}}^{(1)}&{}\Lambda ^{(1)}&{}\cdots &{}0&{}0\\ 0&{} {\mathcal {M}}^{(2)} &{}{\bar{Q}}^{(2)}&{}\cdots &{}0&{}0\\ \vdots &{}\vdots &{}\vdots &{}\ddots &{}\vdots \\ 0&{}0&{}0&{}\cdots &{}{\bar{Q}}^{(C-1)}&{}\Lambda ^{(C-1)}\\ 0&{}0&{}0&{}\cdots &{}{\mathcal {M}}^{(C)}&{}{\bar{Q}}^{(C)}\end{array}\right) , \end{aligned}$$

where ${\bar{Q}}^{(m)}$ is defined as $Q^{(m)}$ with the diagonal entries adapted such that the row sums of *Q* are zero. This means that, in contrast to $Q^{(m)}$, the diagonal entries of ${\bar{Q}}^{(m)}$ depend on *m*.

We conclude that the on/off-seq-*L* process can be seen as a special case of a qbd process. This means that we can use the results in [6] to approximate our likelihood function in a reliable and accurate way. Using the Erlangization technique we can approximate the likelihood ${\mathcal {L}}(\theta \,|\, m^{(k)}_{0,n})$ corresponding to a single data vector $m^{(k)}_{0,n}$ as given in (2), which in turn can be used to approximate the likelihood function (1) corresponding to *N* independent experiments. The maximum likelihood estimate ${\hat{\theta }}$ of $\theta$ can be evaluated by numerical optimization of the likelihood over the domain ${{\mathscr {D}}}$ of $\theta$.

## Results

### Numerical study

In this section we investigate the accuracy of the estimation method for the on/off-seq-*L* process as described above, by means of a simulation-based numerical study. In addition, we identify numerical complications related to the likelihood maximization that we need to take into account, and investigate how to solve them.

Each model setting considered in this section corresponds to a fixed number of phases *L* and to a fixed parameter vector $\theta = (q_{\text {on}}, q_{\text {off}}, \uplambda _1,\ldots ,\uplambda _L, \mu )^\top \in {{\mathscr {D}}}$. In our simulation studies, the model setting and the size of the data were chosen first, by fixing *L* and $\theta$, and fixing *n* and *N*. Next, the data vectors $m_{0,n}^k$, for $k=1,\ldots ,N$, were simulated *B* times, for $B>0$ large and the estimation method was applied to each of the *B* groups of data vectors. Here the parameter $\ell$ in the Erlangization approximation was fixed at $\ell = 2048$ and the domain ${{\mathscr {D}}}$ was chosen as $[0,b]^{L+3}$ for a fixed upper bound $b>0$. This resulted in *B* estimates for the parameter vector $\theta$, which we denote by ${\hat{\theta _i}}$, $i = 1,\ldots ,B$. By analyzing these parameter estimates, we obtained insight in the performance of the estimation method. We performed simulation studies for a variety of model settings and present our findings with the use of a couple of illustrative examples.

#### Imposing constraints

**Fig. 2:**
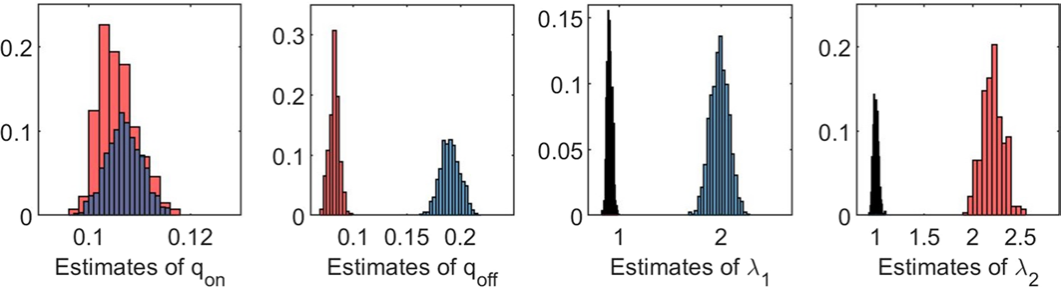
Histograms of 1000 estimates. On/off-seq-2 process with true parameter values: $q_{\text {on}} = 0.1, q_{\text {off}} = 0.2, \uplambda _1 = 2, \uplambda _2 = 1$

The first example concerns the on/off-seq-2 process with parameters $q_{{{\mathrm{on}}}} = 0.1$, $q_{{{\mathrm{off}}}} = 0.2$, $\uplambda _1 = 2$, $\uplambda _2 = 1$ and $\mu = 0$. This means that we start with a model in which only births occur and no deaths, and we consider $\mu$ as a known parameter. Hence, in this example $\theta = (q_{{{\mathrm{on}}}}, q_{{{\mathrm{off}}}}, \uplambda _1, \uplambda _2)^\top$. The size of the data set was fixed, with $n=120$ and $N=375$. The results of a simulation study with $B = 1000$, $b=10$ and $C=100$ are presented in Table 1 and Fig. 2. Table 1 shows, for each parameter, the sample mean of the 1000 estimates and the corresponding sample standard deviation. We observe that the sample means for $q_{\text {off}}$, $\uplambda _1$ and $\uplambda _2$ do not match with the true parameter values, and the corresponding standard deviations are substantial. This is also reflected in Fig. 2, which shows, for each parameter, the histogram of the 1000 estimates. The histograms for $q_{\text {off}}$, $\uplambda _1$ and $\uplambda _2$ clearly consist of two peaks. The estimates corresponding to one parameter vector $\theta$ are displayed in one color, either blue or red, depending on the peak in which the estimate for $q_{\text {off}}$ belongs. It shows that there is a one-to-one relation between peaks of the different parameters. Whenever the estimate for $q_{\text {off}}$ lies in the lower peak (red), the estimate for $\uplambda _1$ lies in the lower peak and the estimate for $\uplambda _2$ lies in the higher peak, and the other way around (blue). We observe that the peaks correspond approximately to the two parameter vectors $\theta _1 = (0.1,0.1,1,2)^\top$ (red), and $\theta _2 = (0.1,0.2,2,1)^\top$ (blue). Note that the blue peaks correspond to the true parameter values of this setting.

**Table 1:** Mean values of 1000 estimates, with corresponding standard deviations. On/off-seq-2 process with true parameter values: $q_{\text {on}} = 0.1, q_{\text {off}} = 0.2, \uplambda _1 = 2, \uplambda _2 = 1$

	${\varvec{q}}_{\textbf{on}}$	${\varvec{q}}_{\textbf{off}}$	${\boldsymbol{\uplambda}}_{\textbf{1}}$	${\boldsymbol{\uplambda}}_{\textbf{2}}$
Mean	0.1066	0.1625	1.7079	1.3115
SD	0.0036	0.0480	0.4780	0.5315

By means of further analysis of the on/off-seq-2 process, we can explain why we find two peaks in Fig. 2. The main reason is that the parameter vectors $\theta _1$ and $\theta _2$ lead to two stochastic processes that are hard to distinguish. This becomes clear by analyzing the distribution of the inter-birth times, the times between consecutive births. Note that these times are i.i.d. We denote the corresponding random variable by *T*. The time between two births always starts in the on-state, and consists of the time it takes to go back and forth between the on- and off-state, and the time it takes to go through the sequential exponential birth phases. Let $G \in \{1,2,\ldots \}$ be a geometrically distributed random variable with parameter $p = \uplambda _1 / (\uplambda _1 + q_{{{\mathrm{off}}}})$, such that $G-1$ can be interpreted as the number of on/off loops of which the inter-birth time *T* consist. Then *T* can be written as the geometric sum

4 $$\begin{aligned} T = \sum _{i=0}^{G-1} A_i + {\tilde{A}}, \end{aligned}$$

where $A_0 = 0$, the $A_i$, for $i\ge 1$, are independent and identically distributed as the sum of two exponential random variables with rates $\uplambda _1 + q_{{{\mathrm{off}}}}$ and $q_{{{\mathrm{on}}}}$, and ${\tilde{A}}$ is distributed as the sum of two exponential random variables with rates $\uplambda _1 + q_{{{\mathrm{off}}}}$ and $\uplambda _2$.

Using expression (4) for *T*, we can study its distribution, starting with the expectation and variance of *T*. Using Wald’s equation on the geometric sum, we see that

$$\begin{aligned} {{{{\mathbb{E}}}}}[T]&= {{{{\mathbb{E}}}}}[G-1] {{{{\mathbb{E}}}}}[A_1] + {{{{\mathbb{E}}}}}[{\tilde{A}}] \\&= \left( \frac{q_{{{\mathrm{off}}}} + \uplambda _1}{\uplambda _1} -1 \right) \cdot \left( \frac{1}{q_{{{\mathrm{off}}}} + \uplambda _1} + \frac{1}{q_{{{\mathrm{on}}}}}\right) + \left( \frac{1}{q_{{{\mathrm{off}}}} + \uplambda _1} + \frac{1}{\uplambda _2}\right) \\&= \frac{1}{\uplambda _1} + \frac{1}{\uplambda _2} + \frac{q_{{{\mathrm{off}}}}}{q_{{{\mathrm{on}}}} \cdot \uplambda _1}. \end{aligned}$$

Similarly, with Wald’s equation for the variance, we find

$$\begin{aligned} {{\,\mathrm{{\mathbb {V}} ar}\,}}[T]&= {{\,\mathrm{{\mathbb {E}}}\,}}[G-1]{{\,\mathrm{{\mathbb {V}} ar}\,}}[A_1] + {{\,\mathrm{{\mathbb {E}}}\,}}[A_1]^2 {{\,\mathrm{{\mathbb {V}} ar}\,}}[G-1] + {{\,\mathrm{{\mathbb {V}} ar}\,}}[{\tilde{A}}] \\&= \left( \frac{q_{{{\,\mathrm{off}\,}}} + \uplambda _1}{\uplambda _1} -1 \right) \cdot \left( \frac{1}{(q_{{{\,\mathrm{off}\,}}} + \uplambda _1)^2} + \frac{1}{q_{{{\,\mathrm{on}\,}}}^2} \right) \\&\quad + \left( \frac{1}{q_{{{\,\mathrm{off}\,}}} + \uplambda _1} + \frac{1}{q_{{{\,\mathrm{on}\,}}}} \right) ^2 \cdot \left( \frac{q_{{{\,\mathrm{off}\,}}}(q_{{{\,\mathrm{off}\,}}} + \uplambda _1)}{\uplambda _1^2} \right) + \frac{1}{(q_{{{\,\mathrm{off}\,}}} + \uplambda _1)^2} + \frac{1}{\uplambda _2^2}\\&= \frac{1}{\uplambda _1^2} + \frac{1}{\uplambda _2^2} + \frac{2q_{{{\,\mathrm{off}\,}}}\uplambda _1 + q_{{{\,\mathrm{off}\,}}}^2 + 2q_{{{\,\mathrm{on}\,}}}q_{{{\,\mathrm{off}\,}}}}{\uplambda _1^2 q_{{{\,\mathrm{on}\,}}}^2}. \end{aligned}$$

**Fig. 3:**
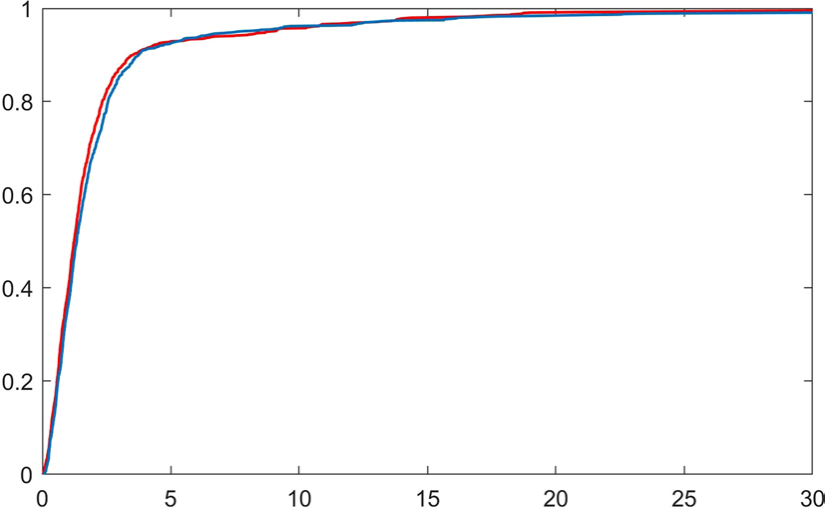
Empirical distribution function of *T* based on 1000 simulated realizations of *T* for parameter vectors $\theta _1$ (red) and $\theta _2$ (blue)

Interestingly, when computing the expectation and standard deviation of *T* for the earlier defined parameter vectors $\theta _1$ and $\theta _2$, we observe almost no difference. Parameter $\theta _1$ gives expectation 2.5 with standard deviation 4.92 and parameter $\theta _2$ gives expectation 2.5 with standard deviation 4.82. This means that, for sample sizes of a realistic size, the distribution of *T* will be indistinguishable for both parameter vectors. This is confirmed by simulations of the distribution of *T*. For both $\theta _1$ and $\theta _2$, $B=1000$ realizations of the inter-birth time *T* were simulated according to (4). Figure 3 shows the corresponding empirical distribution functions for $\theta _1$ in red, and $\theta _2$ in blue. We see that the distribution functions are almost identical, which explains why the two parameter settings $\theta _1$ and $\theta _2$ are indistinguishable, and two peaks appear in Fig. 2.

Intuitively, we can also understand why $\theta _1$ and $\theta _2$ virtually lead to the same stochastic process. Note that in our true parameter setting $\theta _2$, the values for $q_{{{\mathrm{on}}}}$ and $q_{{{\mathrm{off}}}}$ are relatively small compared with the values for $\uplambda _1$ and $\uplambda _2$, hence the phase process dominates the on/off switch. Because of this timescale separation, the time spent in the off-state between two consecutive births is negligible, and the inter-birth time mainly consist of the two exponential phases with parameters $\uplambda _1$ and $\uplambda _2$. Interchanging the two phases will therefore have a modest effect on the inter-birth times, as long as the probability of jumping from state $X_t = 1$ to $X_t = 2$ stays the same. This probability is equal to $\uplambda _1 / (\uplambda _1 + q_{{{\mathrm{off}}}})$, hence if $q_{{{\mathrm{off}}}}$ is adjusted in the right way, the new situation virtually yields the same stochastic process. This is exactly what describes the difference between $\theta _2$ and $\theta _1$. The parameter values for $\uplambda _1$ and $\uplambda _2$ are swapped, and the probability $\uplambda _1 / (\uplambda _1 + q_{{{\mathrm{off}}}}) = 10/11$ in both situations.

**Fig. 4:**
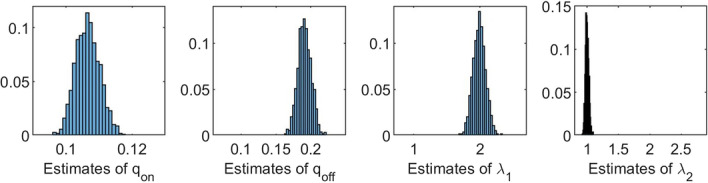
Histograms of 1000 estimates obtained under the constraint $\uplambda _2 \ge \uplambda _1$. On/off-seq-2 process with true parameter values: $q_{\text {on}} = 0.1, q_{\text {off}} = 0.2, \uplambda _1 = 2, \uplambda _2 = 1$

We conclude that for some parameter settings, the shape of the likelihood function is such that numerical maximization can lead to multiple estimates of $\theta$. A way to overcome this numerical complication is by imposing constraints when maximizing the likelihood function. Table 2 and Fig. 4 show the results of a simulation study equal to the one above, with the only difference that the likelihood functions are maximized under the constraint $\uplambda _1 \ge \uplambda _2$, making it no longer possible to interchange $\uplambda _1$ and $\uplambda _2$. We see from Table 2 that the mean values of the 1000 estimates lie close to the true parameter values, and that the standard deviations for the last three parameters decreased considerably. Figure 4 shows us that the histograms of all parameters only have one peak now that we imposed the constraint on $\uplambda _1$ and $\uplambda _2$.

**Table 2:** Mean values of 1000 estimates, with corresponding standard deviations, obtained under the constraint $\uplambda _2 \ge \uplambda _1$. On/off-seq-2 process with true parameter values: $q_{\text {on}} = 0.1, q_{\text {off}} = 0.2, \uplambda _1 = 2, \uplambda _2 = 1$

	$\varvec{q}_{\textbf{on}}$	$\varvec{q}_{\textbf{off}}$	${\boldsymbol{\uplambda}}_1$	${\boldsymbol{\uplambda}}_2$
Mean	0.1066	0.1911	1.9910	1.0004
SD	0.0036	0.0096	0.0960	0.0278

We note that we could also have performed the likelihood maximization under the opposite constraint $\uplambda _1<\uplambda _2$, if for some reason, e.g. biological knowledge of the system at hand, one would have been specifically interested in such candidate solutions. Also, in principle one could first impose the constraint $\uplambda _1 \ge \uplambda _2$ and then the constraint $\uplambda _1<\uplambda _2$, and pick the solution with the highest likelihood; however, in our case those values virtually coincided as a consequence of the two corresponding models being essentially equivalent, and selection of the correct parametersetting is then not guaranteed. The main message is that for some settings of the parameters the corresponding values of the likelihood may be practically indistinguishable, and that without imposing an appropriate constraint on the parameters, the numerical approximation of the maximum likelihood estimate may end up in either one of these parameter settings.

#### The influence of *n* and *N*

In this section we investigate the influence of *n* and *N* on the accuracy of the estimation method. To illustrate our findings, we use the example as above, hence $q_{\text {on}} = 0.1$, $q_{\text {off}} = 0.2$, $\uplambda _1 = 2$, $\uplambda _2 = 1$, with the small adjustment that the death rate of the simulated data, $\mu$, now equals 0.3. Hence, we analyze a model in which both births and deaths occur, and of which the death rate $\mu$ is an unknown parameter as well. Note that the distribution of *T* does not depend on the value of $\mu$, hence we again need to impose the constraint $\uplambda _1 \ge \uplambda _2$ when maximizing the likelihood function.

**Fig. 5:**
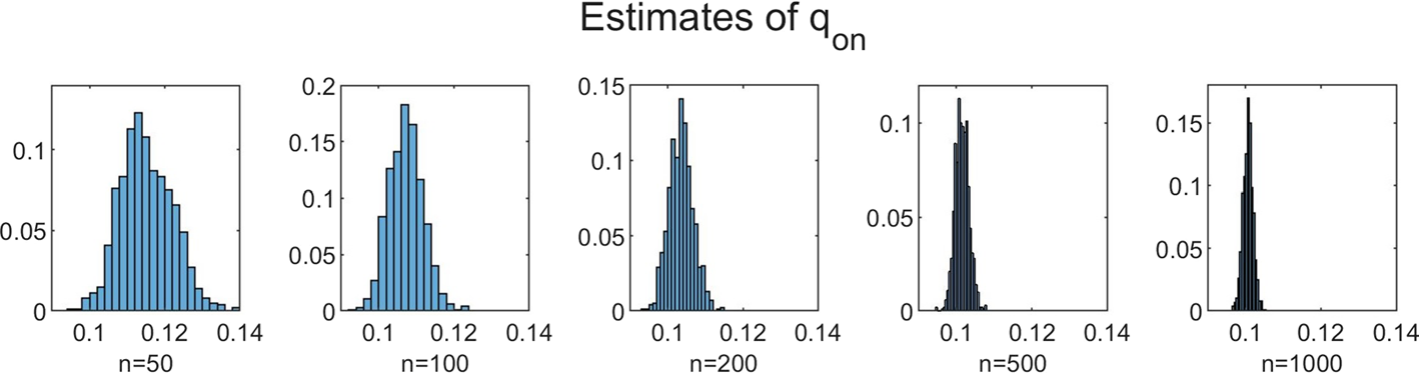
Histograms of the obtained estimates of $q_{\text {on}}$ for increasing values of *n*

**Fig. 6:**
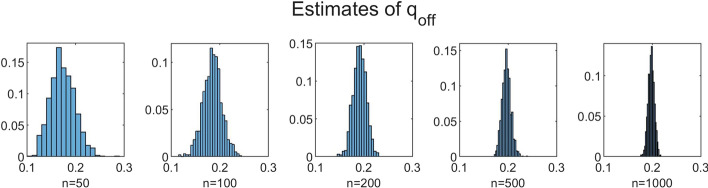
Histograms of the obtained estimates of $q_{\text {off}}$ for increasing values of *n*

**Fig. 7:**
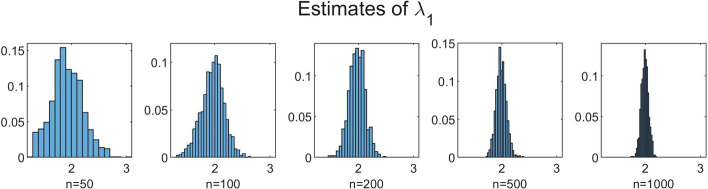
Histograms of the obtained estimates of $\uplambda _1$ for increasing values of *n*

**Fig. 8:**
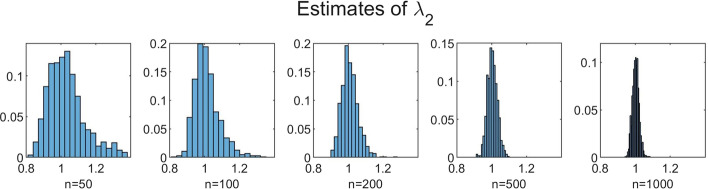
Histograms of the obtained estimates of $\uplambda _2$ for increasing values of *n*

**Fig. 9:**
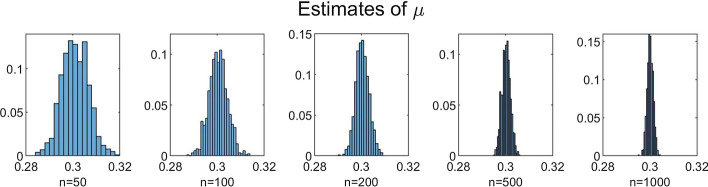
Histograms of the obtained estimates of $\mu$ for increasing values of *n*

To investigate the influence of *n* on the accuracy of the estimation method, we performed simulations for increasing values of *n* with $N = 350$ fixed. We chose $n=50$, $n=100$, $n=200$, $n=500$ and $n=1000$. The results for $B=1000$, $b=10$ and $C=100$ are shown in Table 3 and Figs. 5, 6, 7, 8 and 9. In a few cases, the estimate ${\hat{\theta }}$ ended up at the boundary of the domain ${{\mathscr {D}}}$ over which the likelihood function was maximized. This numerical issue was easily solved by enlarging the domain, after which the estimate ended up in the interior of ${{\mathscr {D}}}$. Table 3 shows, for the increasing values of *n*, the sample mean of the 1000 estimates, with the sample standard deviation between brackets. We see that, for all five parameters, the sample mean lies closer to the true parameter value as *n* increases. Furthermore, the standard deviations decrease as *n* increases. This is also seen in Figs. 5, 6, 7, 8 and 9, which show for each parameter the histograms of the 1000 estimates for the increasing values of *n*. In each figure, the limits of the x-axis are equal for the five histograms, which makes it immediately visible that the histograms become narrower when *n* increases.

**Table 3:** Mean values of 1000 estimates for increasing values of *n* and $N=350$, with corresponding standard deviation between brackets. On/off-seq-2 process with true parameter values: $q_{\text {on}} = 0.1, q_{\text {off}} = 0.2, \uplambda _1 = 2, \uplambda _2 = 1, \mu =0.3$

***n***	${\varvec{q}}_{\textbf{on}}$	${\varvec{q}}_{\textbf{off}}$	${\boldsymbol{\uplambda}}_1$
50	0.1151 (0.0069)	0.1732 (0.0249)	1.9214 (0.2848)
100	0.1072 (0.0045)	0.1847 (0.0190)	1.9607 (0.2063)
200	0.1035 (0.0032)	0.1911 (0.0133)	1.9756 (0.1455)
500	0.1015 (0.0018)	0.1967 (0.0091)	1.9913 (0.0934)
1000	0.1007 (0.0013)	0.1979 (0.0063)	1.9924 (0.0635)

***n***	${\boldsymbol{\uplambda}}_2$	${\boldsymbol{\mu}}$	
50	1.0311 (0.1049)	0.3009 (0.0057)	
100	1.0132 (0.0717)	0.3005 (0.0039)	
200	1.0082 (0.0468)	0.3004 (0.0028)	
500	1.0035 (0.0284)	0.3002 (0.0018)	
1000	1.0023 (0.0194)	0.3001 (0.0013)	

We have seen that the accuracy of the estimation method can be increased by choosing a higher value of *n*. However, in practical situations it is not always possible to increase *n*. This is, for example, the case in the application studied in “RNA transcription” section. One experiment measures the number of RNA molecules in a single cell over time, but the lifetime of a cell is limited. The number of experiments *N*, however, *can* be increased. To investigate the influence of *N* on the accuracy of the estimation method, we performed simulations for increasing values of *N* with $n = 100$ fixed. We considered $N = 200$, $N = 350$, $N = 500$, $N=750$ and $N=1000$. The results for $B=1000$ and $b=10$ are given in Table 4. For each value of *N*, this table shows again the sample mean of the 1000 estimates with the sample standard deviation between brackets. We see that for each parameter, the mean values lie close to the true parameter value, but do not improve as *N* increases. This means that the bias of the estimates is mainly determined by the value of *n*, which is related to how much information is given by one experiment. However, Table 4 also shows that the standard deviations do decrease as *N* increases, and in this way provides insight in how the accuracy increases as a function of *N*.

**Table 4:** Mean values of 1000 estimates for increasing values of *N* and $n=100$, with corresponding standard deviation between brackets. On/off-seq-2 process with true parameter values: $q_{\text {on}} = 0.1, q_{\text {off}} = 0.2, \uplambda _1 = 2, \uplambda _2 = 1, \mu =0.3$

***N***	${\varvec{q}}_{\textbf{on}}$	${\varvec{q}}_{\textbf{off}}$	${\boldsymbol{\uplambda}}_1$
200	0.1072 (0.0059)	0.1849 (0.0252)	1.9642 (0.2679)
350	0.1072 (0.0045)	0.1847 (0.0190)	1.9607 (0.2063)
500	0.1071 (0.0038)	0.1848 (0.0151)	1.9639 (0.1701)
750	0.1072 (0.0031)	0.1850 (0.0124)	1.9627 (0.1376)
1000	0.1072 (0.0027)	0.1849 (0.0106)	1.9609 (0.1176)

***N***	${\boldsymbol{\uplambda}}_2$	${\boldsymbol{\mu}}$	
200	1.0199 (0.0971)	0.3005 (0.0054)	
350	1.0132 (0.0717)	0.3005 (0.0039)	
500	1.0097 (0.0577)	0.3006 (0.0032)	
750	1.0082 (0.0459)	0.3007 (0.0027)	
1000	1.0078 (0.0384)	0.3007 (0.0023)	

#### On/off-seq-3 process

In the first part of the numerical study, we have analyzed the on/off-seq-2 process. In this section we explore the numerical complications related to the likelihood maximization for the on/off-seq-*L* process with $L>2$, and we investigate the accuracy of the estimation method for the on/off-seq-3 process. First note that for $L>2$, the model is partially unidentifiable, since interchanging the parameters $\uplambda _2,\ldots ,\uplambda _L$ yields an identically distributed process $\{M_t\}$. Hence, when performing likelihood maximization, a fixed order of these parameters should be chosen.

The analysis on the inter-birth times of the on/off-seq-2 process can be extended for $L>2$. The inter-birth time *T* can still be written as the geometric sum in (4), but ${\tilde{A}}$ is now distributed as the sum of *L* exponential random variables with rates $\uplambda _1 + q_{{{\mathrm{off}}}}$, $\uplambda _2, \ldots , \uplambda _L$. This means that ${{{{\mathbb{E}}}}}[T]$ and ${\mathbb{V}}{\mathrm{ar}}[T]$ only change by factors $\frac{1}{\uplambda _3} + \cdots + \frac{1}{\uplambda _L}$ and $\frac{1}{\uplambda _3^2} + \cdots + \frac{1}{\uplambda _L^2}$, respectively. We have

$$\begin{aligned} {{{{\mathbb{E}}}}}[T]&= \frac{1}{\uplambda _1} + \cdots + \frac{1}{\uplambda _L} + \frac{q_{{{\mathrm{off}}}}}{q_{{{\mathrm{on}}}} \cdot \uplambda _1}. \end{aligned}$$

Similarly, with Wald’s equation for the variance, we find

$$\begin{aligned} {{{\mathbb{V}} \mathrm{ar}}}[T]&= \frac{1}{\uplambda _1^2} + \cdots + \frac{1}{\uplambda _L^2} + \frac{2q_{{{\mathrm{off}}}}\uplambda _1 + q_{{{\mathrm{off}}}}^2 + 2q_{{{\mathrm{on}}}}q_{{{\mathrm{off}}}}}{\uplambda _1^2 q_{{{\mathrm{on}}}}^2}. \end{aligned}$$

This means that the same reasoning holds as for the on/off-seq-2 process, and additional constraints on $\uplambda _1$ with respect to $\uplambda _2,\ldots ,\uplambda _L$ are needed to make sure that the likelihood function has a unique maximum.

**Fig. 10:**
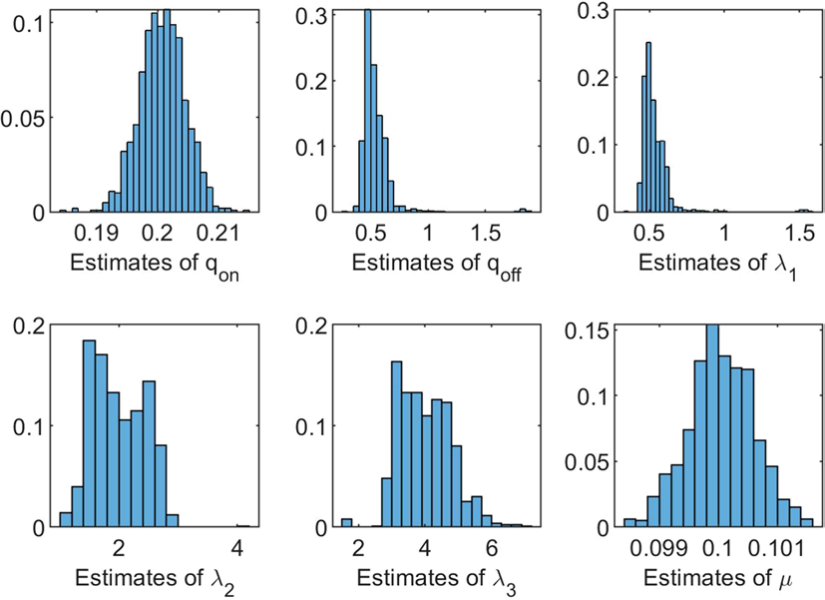
Histograms of 1000 estimates. On/off-seq-3 process with true parameter values: $q_{\text {on}} = 0.2$, $q_{\text {off}} = 0.5$, $\uplambda _1 = 0.5$, $\uplambda _2 = 2$, $\uplambda _3 = 4$, $\mu = 0.1$

To investigate the accuracy of the estimation method for the on/off-seq-3 process, we performed a variety of simulation studies. We present our findings by means of two different examples. The first example is the on/off-seq-3 process with parameters $q_{\text {on}} = 0.2$, $q_{\text {off}} = 0.5$, $\uplambda _1 = 0.5$, $\uplambda _2 = 2$, $\uplambda _3 = 4$ and $\mu = 0.1$. Table 5 and Fig. 10 show the simulation results for this example under the constraint $\uplambda _1 \le \uplambda _2 \le \uplambda _3$, with $B=1000$, $b=10$, $C=100$ and data size $n=1000$, $N=350$. Table 5 shows, for each parameter, the sample mean and corresponding sample standard deviation of the 1000 estimates. We see that the mean values for parameters $q_{{{\mathrm{on}}}}$, $\uplambda _2$, $\uplambda _3$ and $\mu$ lie close to the true parameter values. The mean values for parameters $q_{{{\mathrm{off}}}}$ and $\uplambda _1$, however, exceed the true parameter values. This is also visible in Fig. 10, which shows for each parameter the histogram of the 1000 estimates. The histograms for $q_{{{\mathrm{off}}}}$ and $\uplambda _1$ show some outliers which increase the corresponding means. This example confirms that when *L* increases it becomes more difficult to accurately estimate all model parameters from the data. Hence, as to be expected, for larger *L* more data is needed (i.e. by increasing *n*) to obtain a similar accuracy as for models with a smaller *L*.

**Table 5:** Mean values of 1000 estimates, with corresponding standard deviations. True parameter values: $q_{\text {on}} = 0.2$, $q_{\text {off}} = 0.5$, $\uplambda _1 = 0.5$, $\uplambda _2 = 2$, $\uplambda _3 = 4$, $\mu = 0.1$

	${\varvec{q}}_{\textbf{on}}$	${\varvec{q}}_{\textbf{off}}$	${\boldsymbol{\uplambda}}_1$	${\boldsymbol{\uplambda}} _2$	${\boldsymbol{\uplambda}} _3$	${\boldsymbol{\mu}}$
Mean	0.2008	0.5441	0.5360	1.9895	3.9995	0.1000
SD	0.0037	0.1463	0.1139	0.4370	0.7865	0.0006

**Fig. 11:**
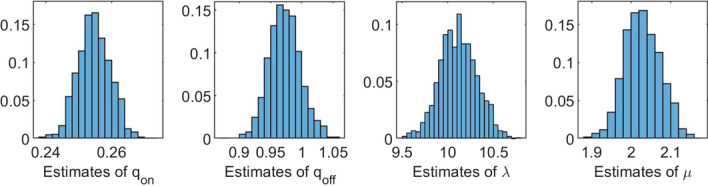
Histograms of 1000 estimates. On/off-seq-3 process with true parameter values: $q_{\text {on}} = 0.25, q_{\text {off}} = 1, \uplambda = 10$ and $\mu = 2$

For some applications it may be more realistic to assume that all $\uplambda _i, i=1,\ldots ,L$, are equal. Under this assumption, the accuracy of the estimation method may increase substantially. We illustrate this by the second example. We consider the on/off-seq-3 process with parameters $q_{{{\mathrm{on}}}} = 0.25$, $q_{{{\mathrm{off}}}} = 1$, $\uplambda _1 = \uplambda _2 = \uplambda _3 = \uplambda = 10$ and $\mu = 2$, hence $\theta = (q_{{{\mathrm{on}}}}, q_{{{\mathrm{off}}}}, \uplambda , \mu )^\top$. The results of a simulation study with $B=1000$, $b=50$, $C=100$, $n=120$ and $N = 375$ are presented in Table 6 and Fig. 11. Table 6 shows, for each parameter, the sample mean and corresponding sample standard deviation of the 1000 estimates. We see that the mean values of the parameters are close to the true parameter values. This is reflected in Fig. 11, which shows for each parameter the histogram of the 1000 estimates. The histograms are nicely shaped around the true parameter values. Note that the size of the data in this example is substantially smaller than in the previous example.

**Table 6:** Mean values of 1000 estimates, with corresponding standard deviations. True parameter values: $q_{\text {on}} = 0.25, q_{\text {off}} = 1, \uplambda = 10$ and $\mu = 2$

	${\varvec{q}}_{\textbf{on}}$	${\varvec{q}}_{\textbf{off}}$	${\boldsymbol{\uplambda}}$	${\boldsymbol{\mu}}$
Mean	0.2547	0.9727	10.1153	2.0282
SD	0.0049	0.0253	0.2028	0.0451

#### Model selection

The estimation method relies on the assumption that the number of phases *L* is known. However, in some situations one would like to select the model that leads to the best representation of the data set. For the on/off-seq-*L* process, this relates to the number of phases *L*, but also to whether an on/off mechanism should be included in the model or not. In this section we investigate a model selection procedure with respect to various on/off-seq-*L* processes. We use the example of “The influence of *n* and *N*” section with $n=100$ and $N=350$, so that the true underlying model is the on/off-seq-2 process with parameters $q_{\text {on}} = 0.1$, $q_{\text {off}} = 0.2$, $\uplambda _1 = 2$, $\uplambda _2 = 1$ and $\mu = 0.3$. We simulated 1000 data sets according to this model. Next we applied the estimation method with respect to six different models, arising from the combination of whether or not there is an on/off mechanism, and if the birth process consists of 1, 2 or 3 phases. This means that next to the on/off-seq-1, on/off-seq-2 and on/off-seq-3 models, we considered the seq-1, seq-2 and seq-3 models in which the on/off mechanism is omitted. For each simulated data set, we computed the six Akaike information criterion (AIC) values from the maximum likelihood estimates corresponding to the six different models under the constraints $\uplambda _1 \ge \uplambda _2$ and $\uplambda _1 \ge \uplambda _2 \ge \uplambda _3$. In 95.7% of the cases the lowest AIC value was indeed found for the correct model, the on/off-seq-2 process. The remaining 4.3% resulted in a lowest AIC value for the on/off-seq-3 process. This outcome confirms the use of the AIC as a sound model selection criterion.

### RNA transcription

In this section we apply the estimation method for the on/off-seq-*L* process, as described at the end of “Quasi birth–death framework” section, to real data of RNA counts. We first describe in detail the biological process of RNA transcription, and then show the results of a model selection procedure that we performed on the data with respect to various on/off-seq-*L* processes.

#### Biological background

**Fig. 12:**
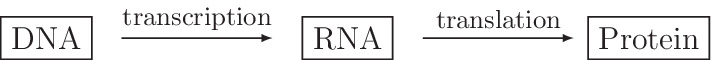
Steps of protein synthesis

Proteins play a major role in the structure and functioning of cells. In fact, all physiological processes in cells depend on proteins. The information needed for the synthesis of proteins is stored in the DNA; think of it as a collection of recipes. Specific parts of the DNA, called genes, contain the information for a particular protein, and can be seen as one recipe. When a protein is needed, the information in the corresponding gene is used for the synthesis of this protein in a process called *gene expression*. Gene expression takes place in two steps, see Fig. 12. In the first step, called *transcription*, the information in the gene is copied into an RNA molecule. In the second step, called *translation*, the copied information in the RNA molecule is used to make the corresponding protein. By transcription, multiple identical RNA molecules can be produced from one gene, and by translation each of these RNA molecules can produce multiple identical proteins. In this way, the proteins can be synthesized with their own efficiency according to the needs of the cell, despite the fact that each cell contains only one or two copies of a specific gene. Interestingly, gene expression is constructed in this way in all cells, from bacteria to humans. We focus on the transcription step in gene expression. It is known that in bacteria the stochasticity in gene expression stems largely from transcription [4], which is why a stochastic model for this process is appropriate.

The transcription of RNA molecules is a complex process. After the transcription of an RNA molecule has been initiated, it takes multiple sequential phases before the molecule is eventually produced. Biologically, RNA transcription takes place through the following steps: first, the molecule RNA polymerase binds to the DNA and slides along the DNA to find a transcription start site, called promoter. Once it has found a start site it binds firmly and the transcription begins. The RNA polymerase moves along the gene while copying the genetical code step by step. Once it reaches the stop site, it releases itself and the new RNA transcript from the DNA. From there, the process can be repeated to produce more RNA molecules. The RNA transcription can be controlled by a process called *gene repression*. The promoter can bind to repressors for a period of time in which RNA polymerase cannot reach the start site to initiate transcription. This causes the promoter to switch between an active state, free from repressors, and an inactive state, bound by repressors.

The on/off-seq-*L* process has been found to be a realistic model for RNA transcription [3, 9], and combines the active/inactive switch of the promoter with the sequential phases of transcription. The phases in the transcription process that contribute to the transcription rate the most are called *rate limiting*, and differ per promoter. Phases that are relatively fast compared to other phases generally do not need to be included in the model. Likewise, it depends on the promoter whether or not the active/inactive mechanism has a (substantial) effect on the transcription dynamics. If the time spent in the inactive state is relatively short compared to the time spent in the active state, it could be decided not to include an on/off mechanism in the model. The model that leads to the best representation of the transcription process can be identified either based on biological considerations or by means of a statistical model selection procedure.

#### Model selection

In this section we describe a model selection procedure that we performed for RNA data corresponding to the so-called $\uplambda$ RM promoter [3], which were kindly provided by prof. A.S. Ribeiro from Tampere University, Finland. The available data set consists of measurements on the number of RNA molecules in a total of 775 single cells, hence $N=775$. Each cell was measured every minute over a period of at most 2 h, depending on the lifetime of the cell, hence $\Delta = 1$ and $n_k \le 121$ (see Remark 1 above). We used the on/off-seq-*L* process to describe the data and applied the Erlangization method as described in “Quasi birth–death framework” section to evaluate the likelihood function and obtain maximum likelihood estimates. As in “Model selection” section, we performed our model selection on six different models, arising from the combination of whether or not there is an on/off mechanism, and if the birth process consists of 1, 2 or 3 phases.

As discussed in “Numerical study” section, imposing constraints on the parameters is an effective way to handle numerical issues regarding local maxima in the approximated likelihood function. Without constraints the numerical maximization may end up in either one of the local maxima. In case of real data one could perform the estimation method under the various constraints and compare the likelihoods corresponding to the solutions, which is what we did. For the on/off-seq-2 process we consider the two cases $\uplambda _1 \le \uplambda _2$ and $\uplambda _2 \le \uplambda _1$. As pointed out in “On/off-seq-3 process” section, for the on/off-seq-3 process, we first need to fix the order of $\uplambda _2$ and $\uplambda _3$, and then consider the various constraints on $\uplambda _1$ with respect to $\uplambda _2$ and $\uplambda _3$. This results in the three cases $\uplambda _1 \le \uplambda _2 \le \uplambda _3$, $\uplambda _2 \le \uplambda _1 \le \uplambda _3$ and $\uplambda _2 \le \uplambda _3 \le \uplambda _1$.

The results of the model selection, with $b=10$ and $C=50$, are shown in Table 7. This table shows for each model/constraint pair the maximum likelihood estimates of the parameters in the first five columns, the sixth column presents the corresponding likelihood values, and the Akaike information criterion (AIC) is shown in the last column. We see that the model that leads to the best fit should contain an on/off mechanism, since the lowest AIC values are found for these models. Within this set of models, the lowest three AIC values are relatively close to each other. We conclude that the on/off-seq-3 process with $\uplambda _2 \le \uplambda _1 \le \uplambda _3$ gives the best fit for this data. However, the on/off-seq-2 process with $\uplambda _2 \le \uplambda _1$ and especially the on/off-seq-3 process with $\uplambda _2 \le \uplambda _3 \le \uplambda _1$ should be considered as plausible options as well. Additional data or biological considerations could help in providing a more conclusive answer.

**Table 7:** Model selection for the $\uplambda$ RM promoter data. The columns show the maximum likelihood estimates, the loglikelihood values, and the AICs, respectively

		${\varvec{q}}_{\textbf{on}}$	${\varvec{q}}_{\textbf{off}}$	${\boldsymbol{\uplambda}} _1$	${\boldsymbol{\uplambda}}_2$	${\boldsymbol{\uplambda}} _3$	${\mathbf{log }}{{\mathcal {L}}}$	AIC
Seq-1	–	–	–	0.0144	–	–	− 3569.9	7141.8
Seq-2	$\uplambda _1 \le \uplambda _2$	–	–	0.0144	8.9245	–	− 3569.4	7142.7
Seq-2	$\uplambda _2 \le \uplambda _1$	–	–	5.8876	0.0144	–	− 3569.5	7143.1
Seq-3	$\uplambda _1 \le \uplambda _2 \le \uplambda _3$	–	–	0.0144	9.9536	9.9875	− 3569.3	7144.6
Seq-3	$\uplambda _2 \le \uplambda _1 \le \uplambda _3$	–	–	5.8876	0.0144	6.0453	− 3570.5	7147.0
Seq-3	$\uplambda _2 \le \uplambda _3 \le \uplambda _1$	–	–	5.8876	0.0144	5.7305	− 3570.7	7147.3
On/off-seq-1	–	0.0249	0.0608	0.0496	–	–	− 3475.2	6956.5
On/off-seq-2	$\uplambda _1 \le \uplambda _2$	0.0303	0.4089	0.2220	0.2221	–	− 3474.8	6957.6
On/off-seq-2	$\uplambda _2 \le \uplambda _1$	0.0254	1.2284	0.7920	0.1416	–	− 3468.2	6944.4
On/off-seq-3	$\uplambda _1 \le \uplambda _2 \le \uplambda _3$	0.0312	0.4410	0.2314	0.2314	9.9983	− 3475.6	6961.3
On/off-seq-3	$\uplambda _2 \le \uplambda _1 \le \uplambda _3$	0.0255	2.4371	1.5558	0.1427	3.8107	− 3466.5	6943.1
On/off-seq-3	$\uplambda _2 \le \uplambda _3 \le \uplambda _1$	0.0254	4.4154	2.8221	0.1423	2.6958	− 3466.9	6943.7

We observe that our findings differ from those in [3]. This can potentially be explained by the fact that by using the constraints we have adequately dealt with possible numerical complications related to likelihood maxima. In addition, as mentioned in the introduction, in [3] the likelihood function is computed from observations of the transcription intervals and not from the RNA counts, where it is noted that these intervals are not known exactly due to the fact that the data is interval-censored.

## Discussion

Motivated by a biological application, we have studied the on/off-seq-*L* process, a bd process with births occurring according to a sequential process consisting of multiple phases and regulated by an on/off mechanism. We have mathematically defined the on/off-seq-*L* process and have shown that it can be seen as a qbd process. The latter enables the use of the Erlangization technique as introduced in [6] to approximate the likelihood function. Maximum likelihood estimates can then be obtained by numerical optimization of this likelihood.

In a numerical study, we have investigated the accuracy of this estimation method for the on/off-seq-*L* process, and have explored numerical complications related to the likelihood maximization. We have shown that for some parameter settings the shape of the likelihood function is such that numerical maximization can lead to multiple estimates of $\theta$. It is therefore necessary to impose constraints on the order of $\uplambda _1, \ldots , \uplambda _L$ when maximizing the likelihood function. Under these constraints, the estimation method works as expected. We have seen that the estimation method yields accurate results, and that the accuracy improves as *n* or *N* increases. As illustrated for $L=3$, the estimation method can also be applied for processes with $L > 2$, but more observations are needed to obtain a similar accuracy as for $L=2$.

We note that the results that we obtained hold for a parameter setting where the phase process dominates the on/off switch. That is, the values for $q_{\text {on}}$ and $q_{\text {off}}$ are relatively small compared to the values for $\uplambda _1,\ldots ,\uplambda _L$. However, parameter settings for which this is not the case should also be explored. Recall that the random variable $G-1$, as in the definition of *T* (4), can be seen as the number of on/off loops of which the inter-birth time consists. Furthermore, ${{\,\mathrm{{\mathbb {E}}}\,}}[G-1] = q_{{{\mathrm{off}}}}/\uplambda _1$, hence the ratio of these two parameters play a major role in how the process behaves. We suspect that there are three different regimes that need to be distinguished with respect to the timescales of the parameters:

- $\uplambda _1$ is substantially higher than $q_{{{\mathrm{off}}}}$. In this case ${{\,\mathrm{{\mathbb {E}}}\,}}[G-1]$ is small and the phase process dominates the on/off switch. This regime corresponds to the settings studied in “Numerical study” section.
- $\uplambda _1$ is substantially smaller than $q_{{{\mathrm{off}}}}$. In this case ${{{{\mathbb{E}}}}}[G-1]$ is large and the on/off switch dominates the phase process. In view of performing statistical inference on the model, this does not seem to be a relevant regime in any practical situation. Only very view births will occur and therefore the on/off mechanism will not be detectable from data on the population size.
- Both $\uplambda _1$ and $q_{{{\mathrm{off}}}}$ are of the same order of magnitude. In view of performing statistical inference on the model, this seems to be a relevant regime when ${{{{\mathbb{E}}}}}[G-1] \le c$, for some constant *c* small enough. At the same time, we expect it to be a complicated regime with its own numerical complications. Preliminary simulation studies suggest that, unless *n* is large, the value of *c* will be hard to distinguish from the data, and hence the corresponding parameters are hard to estimate.

The possible regimes lead us to an important direction for further research. It is interesting to investigate whether there are more relevant regimes and how this can be confirmed mathematically. Moreover, the parameter estimation method should be explored for the last regime, in which all parameters are of the same order of magnitude. Here, one of the questions is whether it is possible to find constraints on the model parameters under which the likelihood maximization will result in accurate estimates.

## Conclusions

The on/off-seq-*L* process is a suitable model for the dynamics of a population of RNA molecules in a single living cell. Analysis of this model can give more insight into the RNA transcription process. The proposed estimation method based on the Erlangization technique is a highly accurate method to find parameter estimates for this model. As expected, the accuracy can be improved by increasing the number of observations *n* or the number of experiments *N*. For larger values of the number of phases *L*, one needs more data in order to obtain an estimate with a given level of accuracy. However, in the situation that all $\uplambda _i$ are equal, the accuracy is substantially better than for models with heterogeneous $\uplambda _i$. Based on experiments in which we apply our estimation method on a real data set of RNA counts, we find empirical backing for the claim that the on/off-seq-3 process is the best model to describe RNA transcription.

## Data Availability

The simulated datasets generated in the context of the current study are available from the corresponding author on reasonable request.
